# Solid state thin electrolyte to overcome transparency-capacity dilemma of transparent supercapacitor

**DOI:** 10.1038/s41598-022-19933-8

**Published:** 2022-09-23

**Authors:** Jongseon Seo, Geonhui Han, Hyejin Kim, Daeseok Lee

**Affiliations:** Department of Electronic Materials Engineering, Kwangwoon University, Seoul, 01897 Republic of Korea

**Keywords:** Condensed-matter physics, Materials for devices, Materials for optics

## Abstract

For portable and transparent electronic applications, transparent supercapacitor (T-SC) is developed to act as an energy storing device. Because electric and optical characteristics of the supercapacitor are strongly dependent on its thickness, all solid state T-SC was developed based on sensitively controllable fabrication process. We were able to attain an optimum thickness for the T-SC such that it exhibited an excellent transparency as well as capacity. Thus, the transparency-capacity dilemma, that is, the thickness of a T-SC increases with respect to its capacity while it is inversely proportional to its transparency, was solved through our proposed T-SC structure. Consequently, more than 60% transparency and 80% capacitance retention of 1500 charge/discharge cycles were achieved. The overcoming of transparency-capacity dilemma can enhance the T-SC applicability as a core energy storage device.

## Introduction

Since the industrial revolution, many studies have been conducted on eco-friendly energy such as reusable wind, solar power, tidal power, and geothermal power^1–4^. For effectively utilize the energy produced in various methods, studies on energy storage device have been actively conducted^5–7^. These energy storage devices are operated electro-chemical reaction by various ion movement, such as Li-ion, Na-ion, K-ion, and etc.^8–16^. Especially, the Li-ion based energy storage device has been studied the most, and commercialization has also been conducted a lot.

In addition, development of portable and transparent electronic gadgets is being extensively researched worldwide, for industrial applications. In this regard, various transparent electronic devices such as a transparent memory, transparent transistors, and transparent displays have been proposed^17–19^. However, for energy storage, transparent energy storing devices need to be further investigated. They need high optical transmittance, high energy/power density, rapid charge/discharge time, and long-term lifetime; transparent supercapacitors have been proposed as one of the energy storing devices^20–23^.

The thickness of a T-SC increases with respect to its capacity while it is inversely proportional to the transparency. In other words, a thin T-SC is required for attaining an optimum transparency, but a high capacity can be achieved using a thick T-SC (transparency-capacity dilemma). In more detail, the self-discharge phenomenon in a thin electrolyte is responsible for the loss of capacity^24^. Thus, it is important to optimize the thickness of the T-SC to overcome the transparency-capacity dilemma.

To overcome these limitations, we developed an all-solid-state T-SC, which can easily manipulate the thickness based on the deposition time. The all solid-state T-SC can also increase the mechanical reliability, reduce the total weight of the device, and exhibit an excellent stability on integration^25^. Consequently, we achieved the T-SC having a transmittance of 60% for visible light and excellent capacitance retention after 1300 charge/discharge cycles.

## Results and discussion

**Figure 1:**
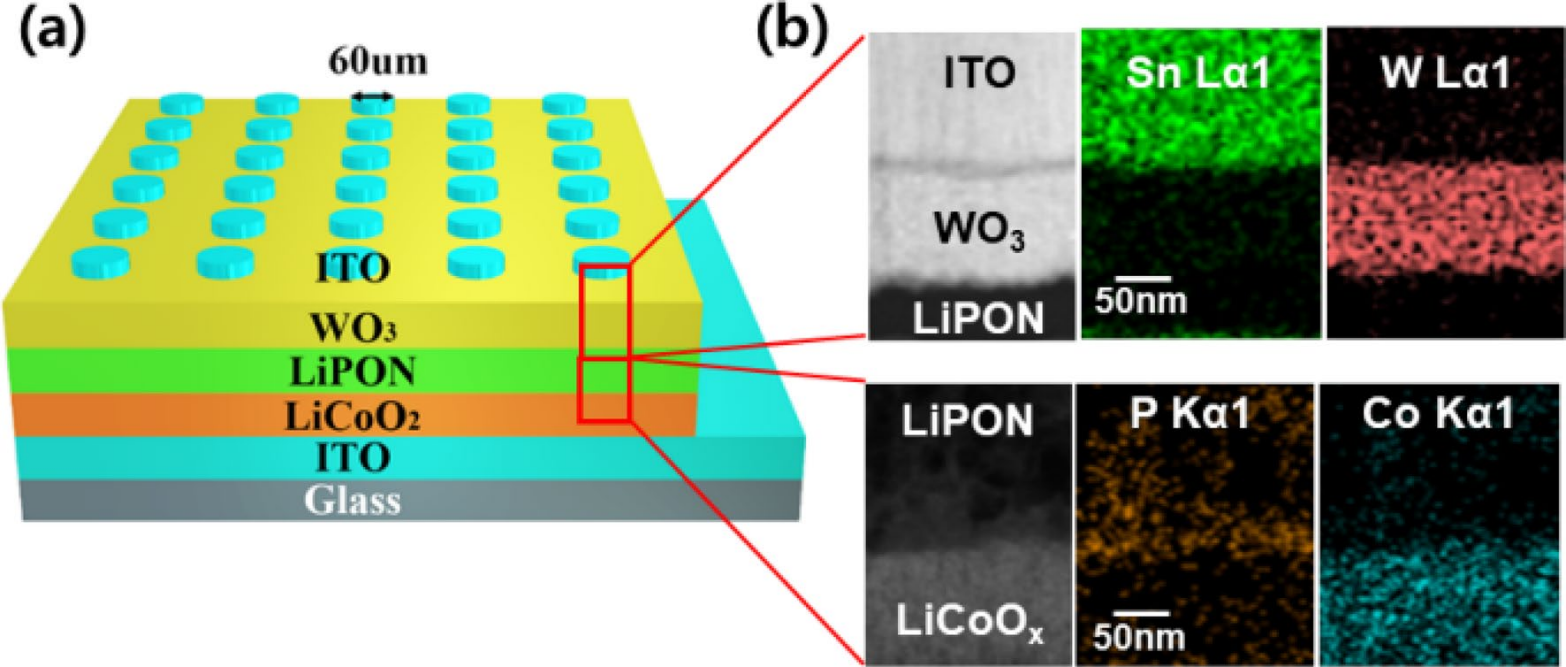
(**a**) Schematics of the developed Glass/ITO/LiCoO$_{2}$/WO$_{3}$/ITO T-SC device. (**b**) Cross-sectional transmission electron microscopy image of the fabricated T-SC device.

Figure 1a illustrates the structure of the developed T-SC. Indium tin oxide (ITO) was used as the material for all electrodes to attain transparency. Furthermore, LiCoO$_{2}$ was used as a cathode layer to provide Li ions during the charging process, and WO$_{3}$ was applied as an anode layer to store the Li ions; LiPON was used as a stable solid-electrolyte materials^26,27^. Figure 1b shows the images of the T-SC obtained through energy dispersive spectroscopy (EDS) elemental mapping and transmission electron microscopy (TEM). The thickness of LiPON was modulated by varying the deposition time. The detailed fabrication process conditions of the optimized device are summarized in “Methods” section and Table 1.

**Table 1:** Optimized T-SC device sputtering process conditions.

Layer	Sputter process			
	Target	Gas (sccm)	Power (W)	Working pressure (mTorr)
LiCoO$_2$ (Cathode)	LiCoO$_2$	Ar 10	100	8
LiPON (Electrolyte)	Li$_3$Po$_4$	Ar/N$_2$ 10/20.5	150	20
WO$_3$ (Anode)	WO$_3$	Ar/O$_2$12/8	150	28.5
ITO (Top electorde)	ITO	Ar 20	100	40

**Figure 2:**
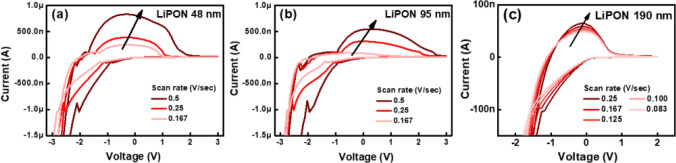
Cyclic voltammetry of three devices with LiPON electrolyte thickness of (**a**) 48 nm, (**b**) 95 nm, and (**c**) 190 nm.

Figure 2a–c show the cyclic voltammetry characteristics for various LiPON thicknesses. These results demonstrate the electrochemical redox reactions between LiCoO$_{2}$ and WO$_{3}$. These redox reactions are expressed as follows^28^.

1 $$\begin{aligned} \begin{array}{ll} LiCoO_{2} \rightarrow Li_{1-x} CoO_{2} + xLi^{+} + xe^{-} \\ WO_{3} + xLi^{+} + xe^{-} \rightarrow Li_{x}WO_{3} \end{array} \end{aligned}$$

**Figure 3:**
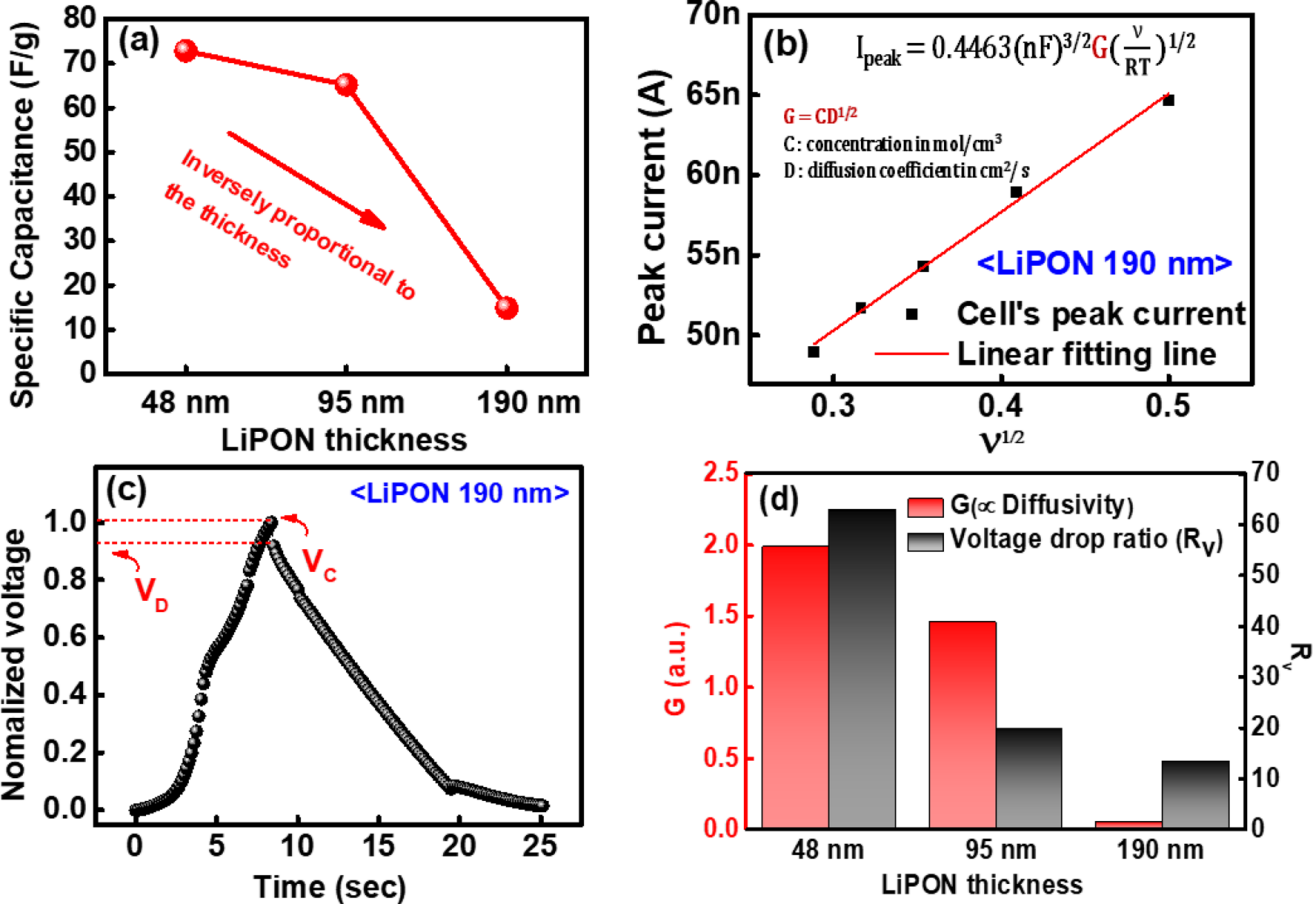
(**a**) Specific capacitance depending on the LiPON thickness. (**b**) Peak current (I$_{peak}$)—scan rate ($\sqrt{\nu }$) curve for various $\nu$, and (**c**) galvanostatic charge discharge curve of LiPON 190 nm T-SC device. (**d**) G (CD$^2$) and voltage drop ratio (R$_{v}$) for different LiPON thicknesses.

Based on this cyclic voltammetry curve, we calculated the specific capacitance according to the following equation.

2 $$\begin{aligned} C_p=\frac{\int IdV}{mv\Delta V} \end{aligned}$$

where $\int$IdV is the area under the cyclic voltammetry curve, $\Delta$V is the potential window, v is the scan rate, and m is the mass of active materials^29^. From the result, we can know that the specific capacitance is inversely proportional to the thickness of LiPON electrolyte layer, as shown in Fig. 3a. Furthermore, we confirmed the movement of Li ions using the Randles-Sevcik equation fitting, as shown in Fig. 3b. The slope of this graph is proportional to G, which is the product of the concentration (C) and the root of diffusivity (D$^{1/2}$). The slope decreases as the thickness of LiPON increases, implying a decrease in diffusivity as well^30^. However, a high diffusivity causes a large amount of desorption of Li ions. As a result, as the LiPON layer became thicker (low diffusivity), the change in peak current (I$_{peak}$) was more stable with the increase in scan rate ($\sqrt{\nu }$). The results of the cyclic voltammetry curve and the galvanostatic charge-discharge curve depict the stability of the developed T-SC.

Figure 3c presents the galvanostatic charge-discharge characteristics of the LiPON 190 nm T-SC device. We also observed the voltage drop characteristic which are responsible for the reduction in power efficiency and capacity. We defined the voltage drop ratio (R$_{v}$), to confirm the drop rate at the maximum voltage as follows:

3 $$\begin{aligned} \text {Voltage drop ratio} (R_{v})= \left[ \frac{V_{C}-V_{D}}{V_{C}} \right] \end{aligned}$$

where V$_{C}$ and V$_{D}$ are the maximum voltages during charging and discharging, respectively (red marked in Fig. 3c). The increase in the desorption of Li ions, owing to a high diffusivity, also increases R$_{v}$. Thus, R$_{v}$ is inversely proportional to the thickness of LiPON. Consequently, it can be observed that G (red bar) and R$_{v}$ (black bar) are inversely proportional to the LiPON thickness, as shown in Fig. 3d.

**Figure 4:**
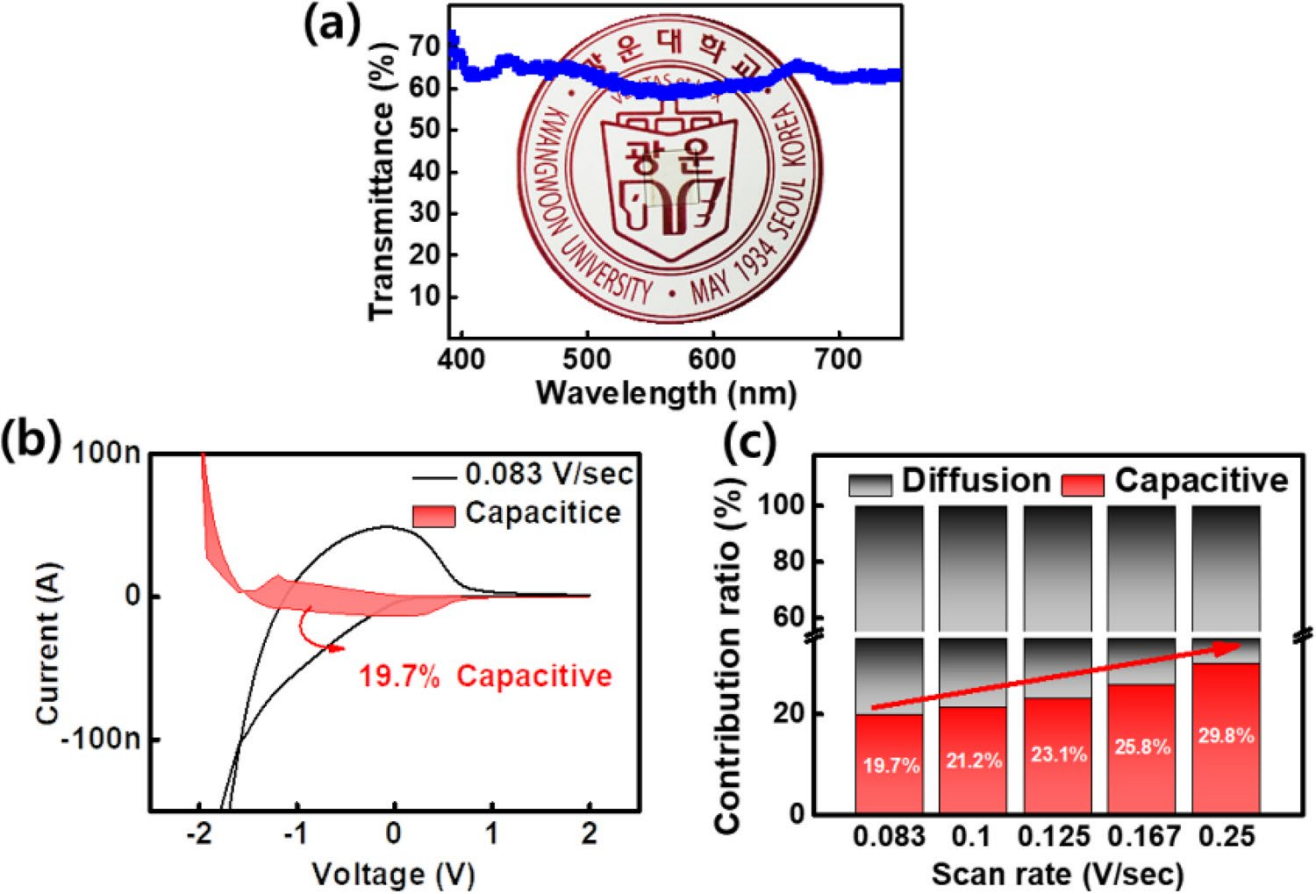
(**a**) Optical transmittance of T-SC device and the corresponding image (inset). (**b**) Capacitive and diffusion-controlled charge storage process at a scan rate of 0.083 V/s. (**c**) Contribution ratio of capacitive and diffusion-controlled currents at various scan rates of the T-SC device.

Thus, we optimized the T-SC device with a 190 nm thick LiPON layer that operates most reliably and exhibits the lowest voltage drop ratio. Figure 4a shows the transmittance (60% or more) characteristics of the T-SC device in the visible light band of 400–750 nm. Additionally, the inset photo also depicts its transparent characteristics. According to the power-law relationship, the ratio of the capacitive current (k$_{1}\nu$) to the diffusion current (k$_{2}\nu ^{1/2}$) in the total current can be expressed as follows :

4 $$\begin{aligned} i(V) =k_{1} \nu + k_{2} \nu ^{1/2} \end{aligned}$$

where i(V) is the current response and k$_{1}$ and k$_{2}$ are constants at different scan rates^31^.

Figure 4b shows that the capacitive current ratio of the T-SC device at a scan rate of 0.083 V/s is 19.7%. The diffusion-controlled and capacitive-controlled contribution percentages for different scan rates are shown in Fig. 4c. The capacitive contribution gradually increases with an increase in the scan rate. Subsequently, it exhibited the highest capacitive current ratio at 0.25 V/s. This indicates the limit of the diffusion process at high scan rates^32^.

**Figure 5:**
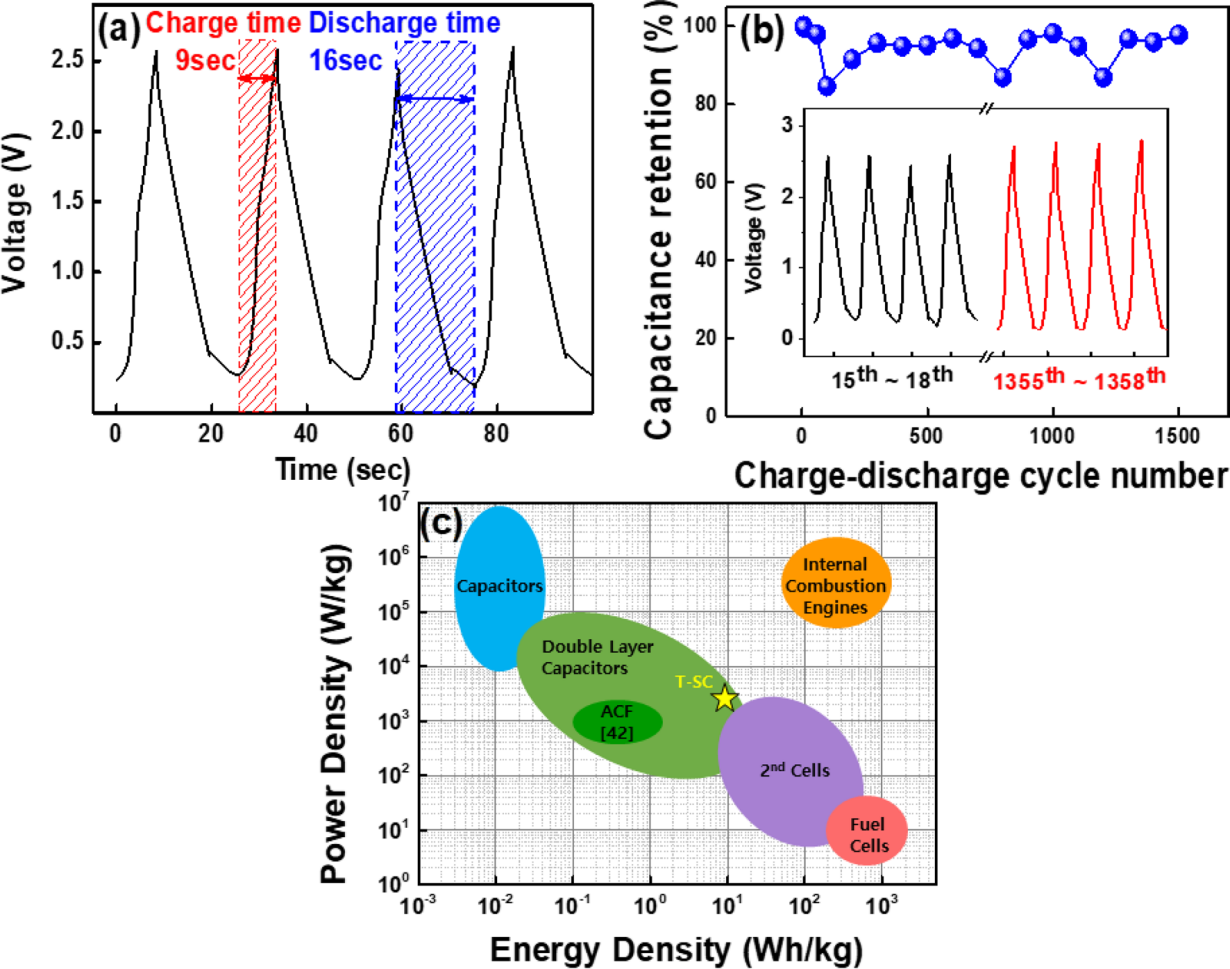
(**a**) Galvanostatic charge discharge curve of the T-SC device. (**b**) Capacitance retention, which denotes a long-term stability of 1500 cycles. (**c**) Ragone plot for various energy storage devices (replotted from Ref.^42^). The developed T-SC device (yellow star) exhibits supercapacitor characteristics.

The galvanostatic charge/discharge curve (17.68 mA/cm$^2$ and 7.072 mA/cm$^2$ of constant charge/discharge current density for 9 and 16 s, respectively.) was approximately triangular with a low voltage drop, as shown in Fig. 5. Moreover, we calculated the coulombic efficiency of the T-SC device based on following equation^33^.

5 $$\begin{aligned} \text {Coulombic efficiency}=\frac{Q_{discharge}}{Q_{charge}}\times 100(\%)= \frac{I_{discharge}\times t_{discharge}}{I_{charge}\times t_{charge}}\times 100 (\%) \end{aligned}$$

where, Q$_{charge}$ and Q$_{discharge}$ are the amount of charge at charging and discharging, I$_{charge}$ and I$_{discharge}$ are the charge, discharge constant current, and t$_{charge}$, t$_{discharge}$ are the charge and discharge time, respectively. As a result, , the T-SC device has a 71.1% coulombic efficiency. In addition, after 1500 charge/discharge cycles, the T-SC exhibited a very stable capacitance (more than 80% of the original capacitance), indicating a long-term electrochemical stability. This is further confirmed by the inconspicuous change between the charging and discharging curves of the 15th–18th and 1355th–1358th cycles, as shown in Fig. 5b. Furthermore, we calculated specific energy density and power density based on below equations:^29,34–36^

6 $$\begin{aligned} E=\frac{1}{2}C_p(\Delta v)^2 \qquad \qquad \qquad P=\frac{E}{\Delta t} \end{aligned}$$

where E is energy density, P is power density, C$_p$ is specific capacitance, $\Delta$v is the voltage scan range, and $\Delta$t is the discharge time. It also exhibited excellent specific energy density of 10.947 Wh/kg and specific power density of 2463.156 W/kg, respectively. From the replotted Ragone plot (fig. 5(c)), the developed device can be considered as the supercapacitor which is located at the intermediate between 2nd cells and capacitor of the Ragone plot.

## Conclusion

We fabricated an all-solid-state T-SC that can be easily optimized and is physically stable. Furthermore, the transparency-capacity dilemma was overcome by controlling the deposition time to regulate the thickness of the electrolyte. Consequently, we fabricated a device with a transmittance of more than 60% transparency in the visible light band. In addition, this device exhibits charge discharge characteristics of up to 1500 cycles and more. It exhibits a high stability during operation with a capacitance retention of at least 80%. Owing to its excellent capacity and transparency characteristics, it is expected to have various applications as a transparent energy storing device.

## Methods

A transparent supercapacitor (ITO/LiCoO$_{2}$/LiPON/WO$_{3}$/ITO) was fabricated on an ITO- coated glass substrate (AMG, Korea). Subsequently, a 100 nm thick layer of LiCoO$_2$ was deposited to act as the cathode, on the 150 nm thick ITO deposited bottom electrode. We deposited LiCoO$_2$ layer by using LiCoO$_2$ target in Ar ambient gas. Sputtering power was 100 W and working pressure was 8 mTorr. Thereafter, a 190 nm thick electrolyte (LiPON) layer was formed by reactive sputtering of a Li$_3$PO$_4$ target in Ar and N$_2$ mixed ambient gas with sputtering power of 150 W at 20 mTorr working pressure. Furthermore, a 100 nm thick (WO$_3$) layer was deposited to act as the anode. The WO$_3$ layer was deposited by utilizing WO$_3$ target in Ar and O$_2$ mixed ambient gas with sputtering power of 150 W and 28.5 mTorr working pressure. Finally, a 150 nm ITO top electrode was deposited using circular shadow mask with a 60 um diameter (in Ar ambient gas with 100 W sputtering power and 40 mTorr working pressure).

Unlike previously reported liquid based fabrication processes^37–41^, to sensitively control the thickness of thin film layer, all layers were fabricated by solid state fabrication processes such as a radio frequency sputtering, reactive sputtering, and photo lithography. In addition, to achieve stable fabrication conditions, commercial sputtering targets purchased from the TAEWON SCIENTIFIC CO. LTD. (with 99.9% purity) were utilized. The detailed fabrication process is summarized as shown in Table 1. The electrical analyses were performed using a Keithley 2450 source-meter.

## Data Availability

The datasets used and/or analysed during the current study available from the corresponding author on reasonable request.
